# Correction to “Identification of 16 novel Alzheimer's disease loci using multi‐ancestry meta‐analyses”

**DOI:** 10.1002/alz.70854

**Published:** 2025-10-24

Willett JDS, Waqas M, Choi Y, et al. Identification of 16 novel Alzheimer's disease loci using multi‐ancestry meta‐analyses. *Alzheimers Dement*. 2025;21(2):e14592. https://doi.org/10.1002/alz.14592. PMID: 39998322; PMCID: PMC11852348.

Throughout our original manuscript, we incorrectly referred to the Alzheimer's Disease Sequencing Project (ADSP) Umbrella dataset as the “NIAGADS dataset.” This dataset should be referred to as the ADSP Umbrella dataset. This dataset includes sequencing data and harmonized phenotypes from cohorts sequenced by the Alzheimer's Disease Sequencing Project and other AD and Related Dementia studies. The National Institute on Aging Genetics of Alzheimer's Disease Data Storage Site (NIAGADS) is the repository hosting the ADSP Umbrella dataset. Additionally, the manuscript did not include the most recent full and correct ADSP acknowledgment statement.

This correction notice serves to clarify these inaccuracies. The authors have updated references to accurately name the ADSP Umbrella dataset as the data source and have updated the full ADSP acknowledgment statement in the Supplementary Material.

We apologize for this error.

